# Acute pancreatitis in a COVID-19 patient in Brazil: a case report

**DOI:** 10.1186/s13256-021-02911-5

**Published:** 2021-10-26

**Authors:** Caroline Petersen da Costa Ferreira, Kalynne Rodrigues Marques, Gustavo Henrique Ferreira de Mattos, Tércio de Campos

**Affiliations:** 1 Hospital Estadual de Francisco Morato Prof. Carlos da Silva Lacaz, Rod. Manoel Silverio Pinto, 125. Belém, Francisco Morato, SP Brazil; 2 Irmandade da Santa Casa de Misericórdia de São Paulo, Rua Cesário Mota Junior 112. Vila Buarque, São Paulo, SP Brazil; 3 Centro Universitário Uninovafapi, Rua Vitorino Orthiges Fernandes, 6123, Planalto Uruguai, Teresina, PI Brazil

**Keywords:** Acute pancreatitis, Pancreatitis in COVID-19, Pancreatic involvement in COVID-19, Case report

## Abstract

**Background:**

The consequences of the coronavirus disease 2019 pandemic have already exceeded 10 million infected and more than 560,000 deaths worldwide since its inception. Currently, it is known that the disease affects mainly the respiratory system; however, recent studies have shown an increase in the number of patients with manifestations in other systems, including gastrointestinal manifestations. There is a lack of literature regarding the development of acute pancreatitis as a complication of coronavirus disease 2019.

**Case report:**

We report a case of acute pancreatitis in a white male patient with coronavirus disease 2019. A 35-year-old man (body mass index 31.5) had acute epigastric pain radiating to his back, dyspnea, nausea, and vomiting for 2 days. The patient was diagnosed with severe acute pancreatitis (AP)-APACHE II: 5, SOFA: 3, Marshall: 0; then he was transferred from ED to the semi-intensive care unit. He tested positive for severe acute respiratory syndrome coronavirus 2 on reverse transcription-polymerase chain reaction, and his chest computed tomography findings were compatible with coronavirus disease 2019. Treatment was based on bowel rest, fluid resuscitation, analgesia, and empiric antibiotic therapy. At day 12, with resolution of abdominal pain and improvement of the respiratory condition, the patient was discharged.

**Conclusion:**

Since there is still limited evidence of pancreatic involvement in severe acute respiratory syndrome coronavirus 2 infection, no definite conclusion can be made. Given the lack of other etiology, we consider the possibility that the patient’s acute pancreatitis could be secondary to coronavirus disease 2019 infection, and we suggest investigation of pancreas-specific plasma amylase in patients with coronavirus disease 2019 and abdominal pain.

## Background

The coronavirus disease 2019 (COVID-19) pandemic affected more than 10 million people and caused more than 560,000 deaths, spreading across 213 countries worldwide, by early July 2020 [1]. COVID-19 is well established as a respiratory tract disease; however, recent studies have shown an increasing number of patients reporting gastrointestinal manifestations such as diarrhea, nausea, vomiting, and abdominal pain [2]. There is a lack of literature regarding the development of acute pancreatitis (AP) as a complication of COVID-19. In this paper, we report a case of acute acalculous pancreatitis in a COVID-19 patient.

The work has been reported in line with the CARE checklist, and it was approved by the local institutional review board.

## Case presentation

A 35-year-old white man was admitted to the emergency department (ED). Two days before admission, he experienced a stabbing epigastric pain radiating to the back and dyspnea as well as nausea and vomiting. Comorbidities included obesity [body mass index (BMI): 31.5] and gastritis treated with omeprazole. He denied allergies and alcohol intake, and did not smoke. He worked as a radiology technician, he had never undergone surgery, and had no health problems in the family.

**Fig. 1:**
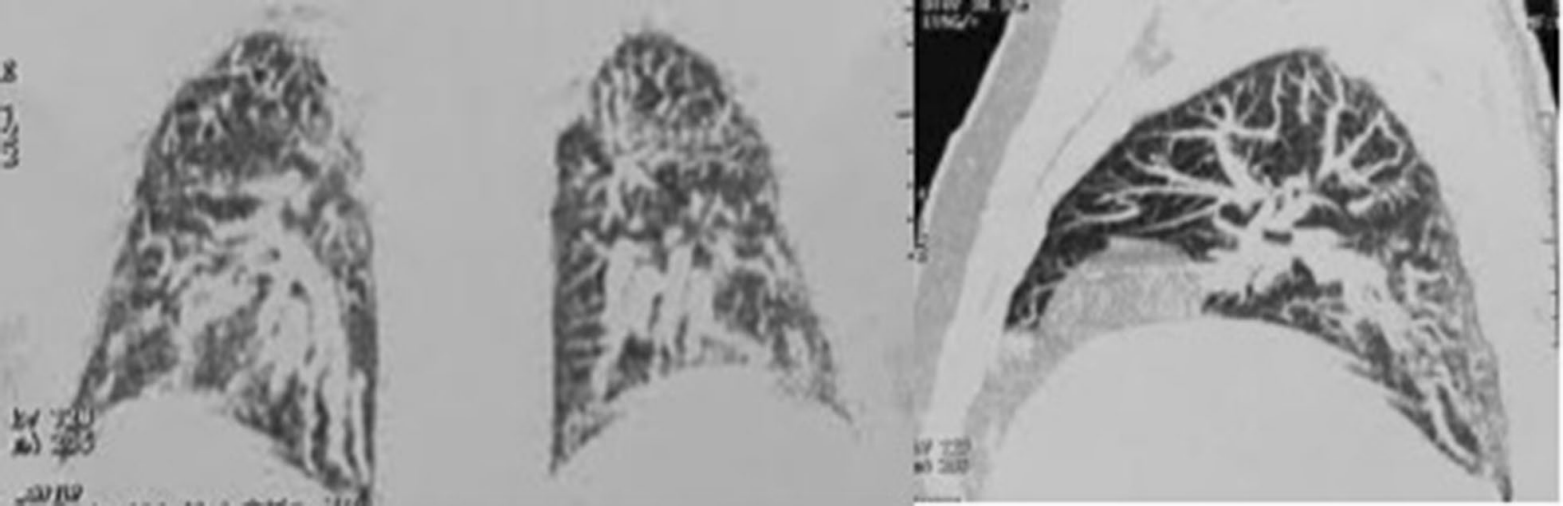
Chest tomography showing multifocal bilateral ground-glass opacities

At admission, the patient had tachycardia (126 beats per minute), normal blood pressure (121 × 95 mmHg), dehydration (+/4+), jaundice (+/4+), and oxygen saturation (SaO_2_) of 95% on room air. Severe epigastric tenderness was noted. There were no other findings on physical and neurological examination. Admission laboratory findings are summarized in Tables 1 and 2. Chest and abdomen computed tomography (CT) both showed multifocal bilateral ground-glass opacities (Fig. 1) and pancreas with increased dimensions and densification of adipose planes in its body and tail, thickening of the left anterior pararenal fascia, minimal amount of free peripancreatic fluid, and normal gallbladder and biliary tract. He had two previous abdominal ultrasounds showing a normal gallbladder.

**Table 1:** Laboratory examinations

Laboratory results	On admission	After 48 hours
White blood cell count (per mm^3^)	13.8	1.57
Band count	5%	20%
Platelet count (per mm^3^)	325.000	139.000
Hemoglobin (g/dL)	16.2	15.8
Alanine aminotransferase (U/L)	297	123
Aspartate aminotransferase (U/L)	217	33
Total bilirubin (mg/dL)	3.6	3.3
Direct bilirubin (mg/dL)	4.6	3.3
Alkaline phosphatase (U/L)	231	121
Urea (mg/dL)	20	44
Creatinine (mg/dL)	0.5	0.9
Amylase (U/L)	1669	
C-reactive protein (mg/L)	1.6	284
Triglycerides (mg/dL)	225	
Calcium (mg/dL)	8.6	
Na	135	140
K	4.5	4.3

**Table 2:** Arterial blood gas analysis

Arterial blood gas analysis	On admission	On the sixth day
pH	7.37	7.43
pCO_2_	33	29
pO_2_	76	64
HCO_3_	20.7	21.7
Base excess	− 5.3	− 4.0
Saturation	95%	93%

The patient was diagnosed with severe AP classified as APACHE II: 5, SOFA: 3, Marshall: 0; then he was transferred from ED to the semi-intensive care unit. Initially, treatment was based on bowel rest, fluid resuscitation, and analgesia with morphine. At day 2, enteral diet was introduced using a post papilla nasoenteral tube. Later, empiric antibiotic treatment for the risk of bacterial pneumonia was started. The patient tested positive for severe acute respiratory syndrome coronavirus 2 (SARS-CoV-2) on reverse transcription-polymerase chain reaction (RT-PCR); then he was transferred to intensive care unit (ICU). A new abdominal ultrasound corroborated a normal gallbladder and biliary tract. Acute hypoxic respiratory failure progressed, and the patient required high-flow oxygen supplementation. Supportive therapy was continued, and, at day 7, oral diet was introduced and the patient showed a gradual resolution of his pulmonary symptoms. The patient spent 9 days in the ICU and was discharged from the hospital 12 days after admission. One month after discharge, the patient was recovering at home, without symptoms. As of the last update, 6 months after discharge, he was fully recovered, without any new AP episode.

## Discussion

The group of coronaviruses (CoV) is a group of single-stranded ribonucleic acid (RNA) viruses, belonging to the subfamily *Orthocoronavirinae* and family *Coronaviridae*, which infect humans and animals. Only the alpha and beta genera can infect humans, with symptoms often associated with a cold [3]. The first epidemic caused by CoV occurred in 2002 in Guangdon, China, by SARS-CoV-1 causing severe acute respiratory syndrome (SARS) with 916 deaths in 29 countries. Then, in 2012, Middle East respiratory syndrome (MERS) caused by a CoV was identified with 858 deaths. In 2019, there were reports of symptomatic individuals who had contact with SARS-CoV-2 present in bats from a wet market in Wuhan, China, being responsible for COVID-19. In the three episodes, there was zoonotic overflow, aggravated by the cultural practices and fragile health laws in those regions [4, 5].

Classical symptoms include fever, cough, and fatigue in addition to sputum, hemoptysis, diarrhea, headache, and lymphopenia. Unique features include targeting of the lower airways and RNAemia, combined with incidence of ground-glass opacity and acute cardiac injury [6].

AP is a disease caused by an abnormality in the activation of pancreatic enzymes with the release of inflammatory mediators, which can compromise peripancreatic tissues and other organs [7]. AP has a multitude of potential causes, including gallstones, alcohol, hypertriglyceridemia, trauma, post-endoscopic retrograde cholangiopancreatography (ERCP), hypercalcemia, medications, anatomic anomalies, and infections or toxins. Known viral causes of pancreatitis include mumps, coxsackievirus, hepatitis B, cytomegalovirus, varicella zoster, herpes simplex, and human immunodeficiency virus [8].

Up to 10% of AP is thought to have an infectious etiology through an immune-mediated inflammatory response, most notably mumps and coxsackie B viruses [9]. However, the pathophysiological mechanism by which a virus, such as the coronavirus, could cause acute pancreatitis is not clear. The main explanations suggest a cytopathic effect of viral replication, or the virus-induced immune response itself [10]. In this patient, other causes of AP were excluded (including alcohol, biliary obstruction/gallstones, anatomic anomalies, drugs, trauma, hypertriglyceridemia, hypercalcemia, and hypotension).

This case demonstrates the possibility of pancreatic injury in patients with COVID-19, in line with previously reported similar cases. A literature review was performed in April 2020 with the following descriptors: acute pancreatitis and COVID-19 in PubMed database, and eight cases were found: two cases in the same family [11], one case in a pregnant woman [12], one in a child [13], one in a woman in Iran [14], one in a woman in Paris [15], one in a woman in the UK [16], and one in a man in Romania [17]. Among these eight cases, only one case occurred in an adult man [17], as in the case described in this report.

A new literature review was carried out in May 2021 with the following descriptors: acute pancreatitis and COVID-19 in PubMed database, and about 90 papers showed up. Of these, 54 were case or series reports. One study shows the point prevalence, risk factors, and outcomes of 32 hospitalized patients with COVID-19 presenting with acute pancreatitis in a large health system and compares outcomes of pancreatitis in patients without COVID-19 [18]. Another one analyzed the clinical profiles of 17 patients with COVID-19 and acute pancreatitis [19]. Most of the other studies were about the increase of amylase and lipase without pancreatitis or about the possible relationships between these two entities. Further large studies are needed to confirm these findings. A better understanding of the relationship between AP and COVID-19 will guide clinicians on early management strategies and focus medical resources toward those patients at risk for worse outcomes.

The increased interest in studying the relationship between AP and COVID-19 suggests a yet to be delineated complex interaction between them.

It is important to note that patients infected with COVID-19 can present the less common gastrointestinal symptoms without early respiratory symptoms. This highlights the need for appropriate personal protective equipment for providers, even when COVID-19 is not initially on the differential diagnosis.

## Conclusion

Given the lack of other etiology, we consider the possibility that the patient’s acute pancreatitis could be secondary to COVID-19 infection, and we suggest investigation of pancreas-specific plasma amylase in patients with COVID-19 and abdominal pain.

## Data Availability

Not applicable.

## Abbreviations

CT: Computed tomography
AP: Acute pancreatitis
CoV: Coronaviruses
ERCP: Endoscopic retrograde cholangiopancreatography
ICU: Intensive care unit
SARS: Severe acute respiratory syndrome
